# Supplementary figures and images for: Timing of Transcriptomic Peripheral Blood Mononuclear Cell Responses of Sheep to Fasciola hepatica Infection Differs From Those of Cattle, Reflecting Different Disease Phenotypes
Source: Front Immunol. 2021 Sep 20;12:729217. doi: 10.3389/fimmu.2021.729217 (PMC8488161; doi:10.3389/fimmu.2021.729217)

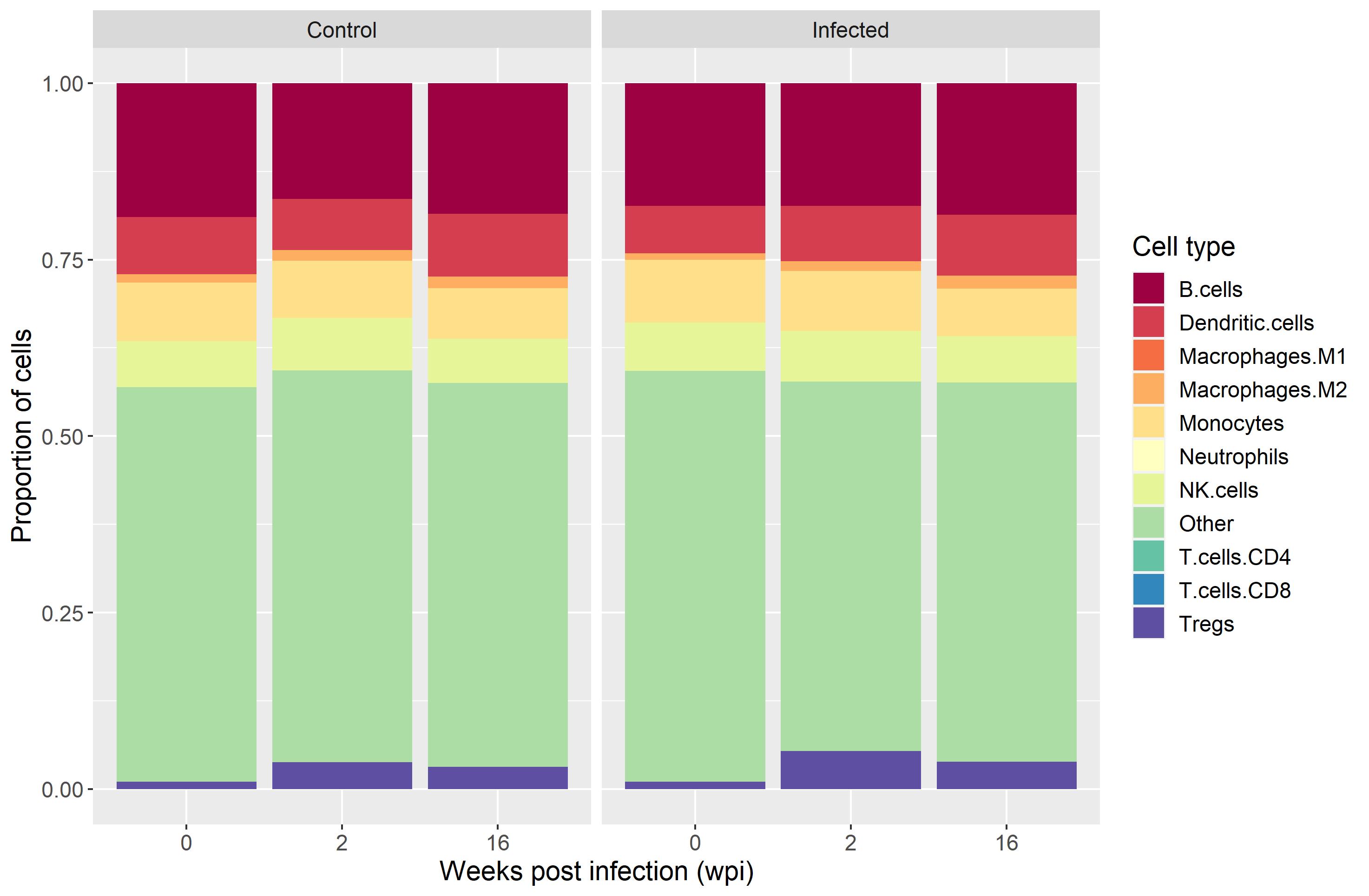

Supplement: Supplementary Figure 1 — Changes in cellular composition in PBMC infected and control groups over time illustrated by cell proportions. [file DataSheet_1.zip › Supplementary_Figure_1_ovine_PBMC_transcriptomics_Fhepatica.jpeg]
